# Cost-Effectiveness of Double Reading versus Single Reading of Mammograms in a Breast Cancer Screening Programme

**DOI:** 10.1371/journal.pone.0159806

**Published:** 2016-07-26

**Authors:** Margarita Posso, Misericòrdia Carles, Montserrat Rué, Teresa Puig, Xavier Bonfill

**Affiliations:** 1 Service of Clinical Epidemiology and Public Health, Biomedical Research Institute Sant Pau (IIB Sant Pau), Barcelona, Spain; 2 Economics Department, Universitat Rovira i Virgili, Reus, Spain; 3 Basic Medical Sciences Department, Biomedical Research Institut of Lleida (IRBLLEIDA), Universitat de Lleida, Lleida, Spain; 4 Universitat Autònoma de Barcelona (UAB), Barcelona, Spain; 5 CIBER of Epidemiology and Public Health (CIBERESP), Barcelona, Spain; State University of Maringá/Universidade Estadual de Maringá, BRAZIL

## Abstract

**Objectives:**

The usual practice in breast cancer screening programmes for mammogram interpretation is to perform double reading. However, little is known about its cost-effectiveness in the context of digital mammography. Our purpose was to evaluate the cost-effectiveness of double reading versus single reading of digital mammograms in a population-based breast cancer screening programme.

**Methods:**

Data from 28,636 screened women was used to establish a decision-tree model and to compare three strategies: 1) double reading; 2) double reading for women in their first participation and single reading for women in their subsequent participations; and 3) single reading. We calculated the incremental cost-effectiveness ratio (ICER), which was defined as the expected cost per one additionally detected cancer. We performed a deterministic sensitivity analysis to test the robustness of the ICER.

**Results:**

The detection rate of double reading (5.17‰) was similar to that of single reading (4.78‰; P = .768). The mean cost of each detected cancer was €8,912 for double reading and €8,287 for single reading. The ICER of double reading versus single reading was €16,684. The sensitivity analysis showed variations in the ICER according to the sensitivity of reading strategies. The strategy that combines double reading in first participation with single reading in subsequent participations was ruled out due to extended dominance.

**Conclusions:**

From our results, double reading appears not to be a cost-effective strategy in the context of digital mammography. Double reading would eventually be challenged in screening programmes, as single reading might entail important net savings without significantly changing the cancer detection rate. These results are not conclusive and should be confirmed in prospective studies that investigate long-term outcomes like quality adjusted life years (QALYs).

## Introduction

Mammogram is the test of choice in European breast cancer screening programmes since it can detect breast cancer at an early stage [1–3]. Whereas digital mammography is a technology that can reduce false-positive results, no significant differences in the cancer detection rate were stated when it was compared to screen-film mammography [4]. In addition, an evaluation of its costs showed that screening with digital mammography can save long-term budget expense in breast cancer screening programmes [5]. Screening with digital mammography, therefore, has been widely implemented.

As two readers are unaware of each other’s interpretation, double reading can increase sensitivity reducing the chance of missed lesions [6–12]. Thus, double reading of digital mammograms became the usual practice in European programmes [1]. However, the following reasons might bring into question its cost-effectiveness.

The effectiveness of double reading may be less important in situations where a high level of agreement between radiologists exists [13]. The benefit of double reading may be restricted to particular settings in which cancer detection is difficult, i.e. mammograms of women in their first participation (prevalent screening) when no previous images are available, women with small lesions that are not easy to find, or when the readers are less experienced [6,14–17]. In addition, having two readers may significantly increase the time, staff costs and resources used in the reading process [18].

Information obtained from cost-effectiveness analyses is useful to decision makers when deciding to implement breast cancer screening programmes and evaluate its benefits and potential harms. One previous cost-effectiveness analysis based on European data, reported that risk-based strategies could reduce harms and costs [19]. Shifting from double reading to single reading was not analysed in this study. However, it is reasonable to hypothesize that in some contexts more benefits can be obtained from single reading as it may reduce costs and false-positives without significantly reducing the cancer detection rate [20]. Conversely, other cost-effectiveness analyses performed in European countries reported double reading as a cost-effective strategy in programmes that used screen-film mammography [1,6,21,22],

Recently, economic evaluations have focused on the cost-effectiveness of double reading versus the combination of single reading and CAD (computer-aid detection) [23–26], whilst studies of single reading without CAD have not yet been published in the context of digital mammography [23,27]. In fact, as further as we know, little is known about whether double reading is a cost-effective strategy in digital screening. Therefore, the main purpose of this study was to assess the cost-effectiveness of double reading versus single reading of digital mammograms in a breast cancer screening programme.

## Materials and Methods

### Study population

This study was performed in women participating in a population-based breast cancer screening programme of the Hospital Sant Pau in an area of 390,000 inhabitants in Barcelona, Spain. The programme was funded by the Public Health Insurance to invite women of 50–69 years of age for biennial screening. In this study, we included all digital mammograms performed from June 2009 until May 2011 and followed up until May 2013. We excluded two mammograms because the data of the reader’s interpretation was incomplete. A total of 28,636 mammograms were analysed, 5,978 (20.9%) corresponding to women participating for the first time in the breast cancer screening programme (prevalent screening), and 22,658 (79.1%) corresponding to subsequent participations (subsequent screening) (Table 1).

**Table 1:** Characteristics of the women included in the analysis.

	Participants in one screening round (2009–2011)	
	No.	%
**Study population**^**¶**^	28,636	100.0
Prevalent screening	5,978	20.9
Incident screening	22,658	79.1
**Age at screening**		
50–54	8,181	28.6
55–59	6,947	24.3
60–64	7,047	24.6
65–69	6,461	22.6

^¶^The information of these women was included in the decision-tree model as common parameters for all reading strategies.

**Fig 1:**
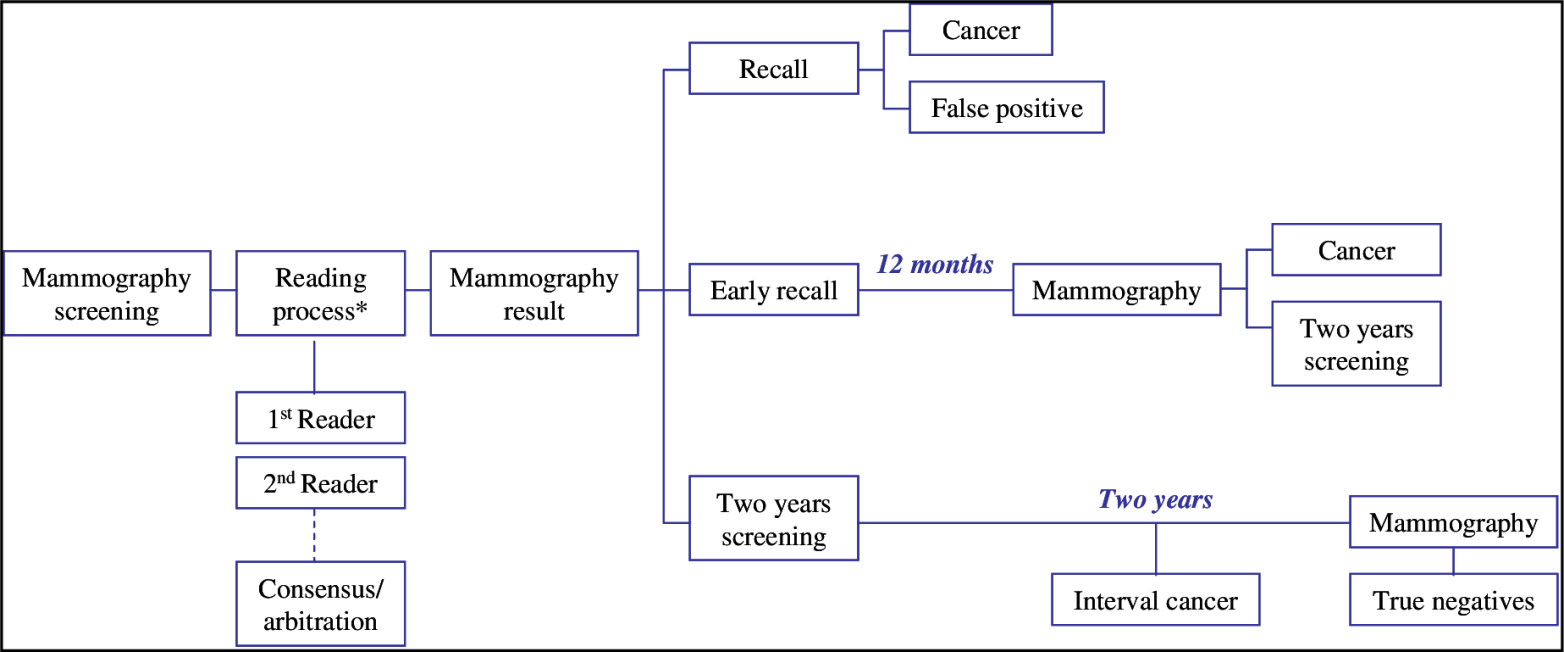
Algorithm followed during a biennial screening round in the programme. *The reading process included independent double reading followed by consensus and arbitration in case of disagreement.

Two projections (mediolateral-oblique and craniocaudal) were taken per breast in all screening examinations. Four certified screening radiologists, who read at least 5,000 mammograms per year, read the mammograms in the breast cancer screening programme. Two radiologists separately read each mammogram (independent double reading). The radiologists classified the results of each mammogram as follows: (1) recall, in which case additional tests were requested to confirm or ruled out malignancy; (2) early recall, in which case the woman was programmed for a further screening mammography in 12 months; or (3) two-year screening, in which case the woman was programmed for a further screening mammography in two years. In case of disagreement between radiologists, the result of the mammogram was determined by consensus or by arbitration. Fig 1 shows the algorithm of the decisions made in a screening round.

Screen-detected cancers were pathologically confirmed breast cancers, both ductal carcinoma in situ (DCIS) and invasive carcinomas. We did not include breast malignancies other than primary breast cancers. The cancer detection rate was calculated as the number of screen-detected cancers divided by the number of participants. The Ethics Committee of the Hospital Sant Pau approved the study. Informed consent was not required, since the data were retrospectively collected, and records were anonymized before receipt and analysis.

### Model design

A decision-tree model was used because of its appropriateness to reflect the immediate effects of decisions taken during a screening round. In this model, the main outcome of effectiveness was the screen-detected cancers; other performance and diagnostic accuracy outcomes were also analysed (S1 Table). The time horizon was four years–from June 2009 to May 2013–, which included a biennial screening round (2009–2011) plus two-year follow-up to confirm negative results. This model assumes that participants do not suffer other conditions that prevent them from successfully completing the entire time horizon.

**Fig 2:**
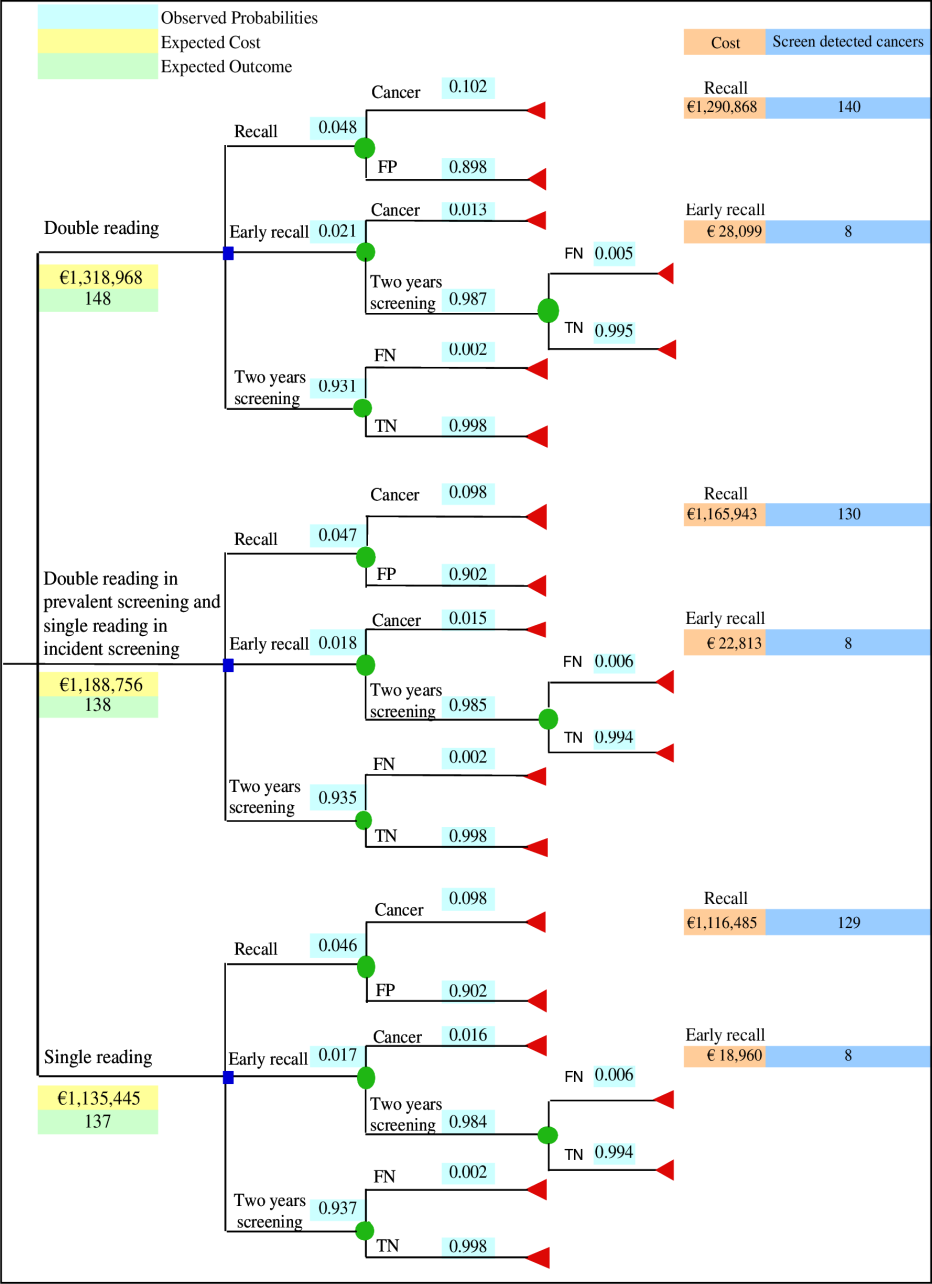
Decision-tree model used to evaluate the cost-effectiveness of the three reading strategies. FP = False Positive. FN = False Negative. TN = True Negative.

The cost-effectiveness of double versus single reading was evaluated comparing the following strategies (Fig 2):

#### Double reading

Double reading with consensus and arbitration was the procedure performed in the breast cancer screening programme. For this strategy, we included the results of mammograms (recall, early recall or two-year screening) in the model, as well as the number of cancers in the way they were observed in a real setting.

#### Double reading in prevalent screening and single reading in incident screening

This strategy was based on the assumption that double reading was performed only for mammograms of women in their first participation, and single reading was performed for mammograms of women in their subsequent participations.

#### Single reading

This strategy was based on the assumption that the first radiologist alone determined the result of mammograms. In order to evaluate the effect of switching from the first to the second reader, we included the results of the second radiologist in the sensitivity analysis.

### Costs data

As this study was conducted from the perspective of the public health system, we included direct healthcare costs of practitioner and diagnostic tests. Total costs were calculated using a bottom-up costing method in two stages: mammography screening and additional tests. Unitary costs for the first stage were obtained from the programme database. The financial department at the hospital provided costs for the second stage. All costs were calculated in Euro (€) (2010 value) (S2 Table). Since long-term health benefits are expected in a preventive programme, NICE’s recommendation carries a lower discount rate that reflects society’s preferences for benefits in the future [28–30]. In this study, we used a short-term horizon that prevents important variations associated with discounting. For these two reasons, no discount rate has been applied.

### Determining cost-effectiveness

We compared reading strategies using incremental cost-effectiveness ratio (ICER) that indicates the additional cost of obtaining one additionally screen detected cancer. To construct the hierarchy of cost-effectiveness of the reading strategies we did the following: firstly, we calculated the average cost-effectiveness ratio (ACER) by dividing the total costs of each strategy by its corresponding number of screen detected cancers. This allowed us to ruled out, when necessary, a dominated strategy (more costly and less effective than alternatives). Secondly, we calculated ICERs and reported them starting with the lowest additional cost per additionally detected cancer. This allowed us to identify a strategy with lower effectiveness and higher ICER and to rule it out for extended dominance. Finally, ICERs for the non-ruled out strategies were compared. The most cost-effective strategy showed the lowest ICER.

### Sensitivity analysis

A deterministic sensitivity analysis was carried out to assess the robustness of ICERs according to the variation in the following variables: participation rate, breast cancer prevalence, sensitivity of the reading strategy, positive-predictive value (PPV) of recall, early recall rate and staff costs (S3 Table).

Statistical tests were two-sided and all P values of less than 0.05 were considered statistically significant. We used Microsoft Excel, Redmond, Washington (2011) in all analyses.

## Results

### Effectiveness of reading strategies

The characteristics of the 28,636 women included in the study are presented in Table 1. The most relevant data for comparing the three reading strategies are the following. The number of screen detected cancers of the three strategies was: 148 (5.17‰) for double reading, 138 (4.82‰) for double reading in prevalent screening and single reading in incident screening, and 137 (4.78‰) for single reading (P = 0.768). Sixteen interval cancers (0.56‰) occurred after double reading (Table 2).

**Table 2:** Effectiveness-outcomes of reading strategies included in the model.

	Double reading		Double reading in prevalent screening and single reading in incident screening		Single reading		P value
	No.	%	No.	%	No.	%	
**Mammogram results**							
Recall	1,366	4.8	1,333	4.7	1,322	4.6	.004
Early recall^†^	609	2.1	523	1.8	490	1.7	
Two-year screening	26,661	93.1	26,780	93.5	26,824	93.7	
**Performance measure**							
Readers’ agreement	27,022	94.4	27,982	97.7	NA	NA	< .001
Consensus or arbitration	1,614	5.6	654	2.3	NA	NA	
PPV of recall	140	10.2	130	9.8	129	9.8	.884
**Diagnostic accuracy**							
Sensitivity	148	94.8%	138	88.5%	137	87.8%	.027^∑^
Specificity	27,214	95.5%	27,242	95.6%	27,256	95.7%	.389^∑^
**Cancers**							
Cancer detection rate^¶^	148	5.17‰	138	4.82‰	137	4.78‰	.768
Interval cancers^§^	16	0.56‰	26	0.91‰	27	0.94‰	.200 .093^∑^
**Histologic type**							
Invasive	116	79.5	111	81.6	111	82.2	.822
In situ	30	20.5	25	18.4	24	17.8	
Unknown	2	-	2	-	2	-	

NA = not applicable. PPV = positive predictive value.

^†^Women referred to mammography-control in 12 months.

^∑^Chi square test of double versus single reading.

^¶^Detection rate per 1000 screened women.

^§^Interval cancers detected in the subsequent two years after a negative screening episode.

#### Characteristics of screen detected cancers

The number of screen detected cancers at double reading was higher in incident screening (n = 104) than in prevalent screening (n = 44). However, compared to incident screening (4.59‰), the cancer detection rate was higher in prevalent screening (7.36‰; P = 0.008). Of the 148 screen detected cancers, 117 (79.1%) were detected in concordance between both radiologists, while 31 (20.9%) were detected after consensus or arbitration. Thirty (20.5%) were carcinomas in situ (CIS) and 116 (79.5%) were invasive carcinomas. Compared to single reading, double reading increased by 25% (n = 6) and 4.5% (n = 5) the number of CIS and invasive carcinomas, respectively. No other relevant differences were observed in the characteristics of screen detected cancers according to reading strategies (S3 Table).

### Cost of reading strategies and additional diagnostic tests

The cost of the three strategies was the following: €1,318,968 for double reading; €1,188,756 for double reading in prevalent screening and single reading in incident screening; and €1,135,445 for single reading. The final amount of common costs was €855,298, which was 64.9% and 75.3% of the total costs at double and single reading, respectively. Differences in non-common costs were mostly attributed to the number of readings and additional diagnostic tests (Table 3).

**Table 3:** Costs estimation for a biennial period (2009–2011) according to reading strategy.

	**Common parameters for all three reading strategies**						
	**No.**				**Cost €**		
Mammogram	28,636				164,787		
Staff	NA				690,510		
**Non-common costs**	**Double reading**		**Double reading in prevalent screening and single reading in incident screening**			**Single reading**	
	**No.**	**Cost €**	**No.**	**Cost €**		**No.**	**Cost €**
^ß^Staff	NA	255,363	NA	156,874		NA	123,052
^¶^Additional tests	1,414	205,170	1376	173,890		1361	154,570
Supplies in early recall	609	2,331	523	2,002		490	1,876
*Maintenance in early recall	NA	807	NA	693		NA	649
**Total**	**1,318,968**		**1,188,756**			**1,135,445**	

NA = not apply.

^ß^Radiologist and administrative staff involved in the reading process, consensus or arbitration; also administrative and technical staff involved in the early recall process.

^¶^Ultrasound, additional mammography, fine-needle aspiration cytology, core biopsy, open surgical biopsy, other minimal procedures.

*Including depreciation of mammography machine.

### Cost effectiveness analysis

#### Average cost-effectiveness ratio

The average cost of each screen detected cancer for the three strategies was €8,912 for double reading; €8,614 for double reading in prevalent screening and single reading in incident screening; and €8,287 for single reading. Compared to double reading, the other two strategies were less expensive and less effective; therefore, none of the strategies was ruled out because of dominance.

#### Incremental cost-effectiveness ratio (ICER)

Table 4 shows the process to calculate ICERs. The ICER of double reading in prevalent screening and single reading in incident screening versus single reading was €53,312. This strategy was ruled out of the analysis due to extended dominance. The ICER of double versus single reading was €16,684. This amount was more than twice the average cost per one cancer detected at single reading (€8,287).

**Table 4:** Process to calculate the Incremental Cost-Effectiveness Ratio (ICER) of the reading strategies.

Reading strategy	Expected cost €	Expected outcome		Incremental cost €	Incremental effect	Average cost €	ICER €
		No. of cancers	Detection rate				
Single reading	1,135,445	137	4.78‰			8,287	
Double reading in prevalent screening and single reading in incident screening	1,188,756	138	4.82‰	53,312	1	8,614	53,312
Double reading	1,318,968	148	5.17‰	183,523	11	8,912	^**¶**^**16,684**

^**¶**^The strategy that combines double reading in prevalent screening with single reading in incident screening was ruled out by extended dominance; therefore, the value of the ICER represents the comparison between double and single reading.

**Fig 3:**
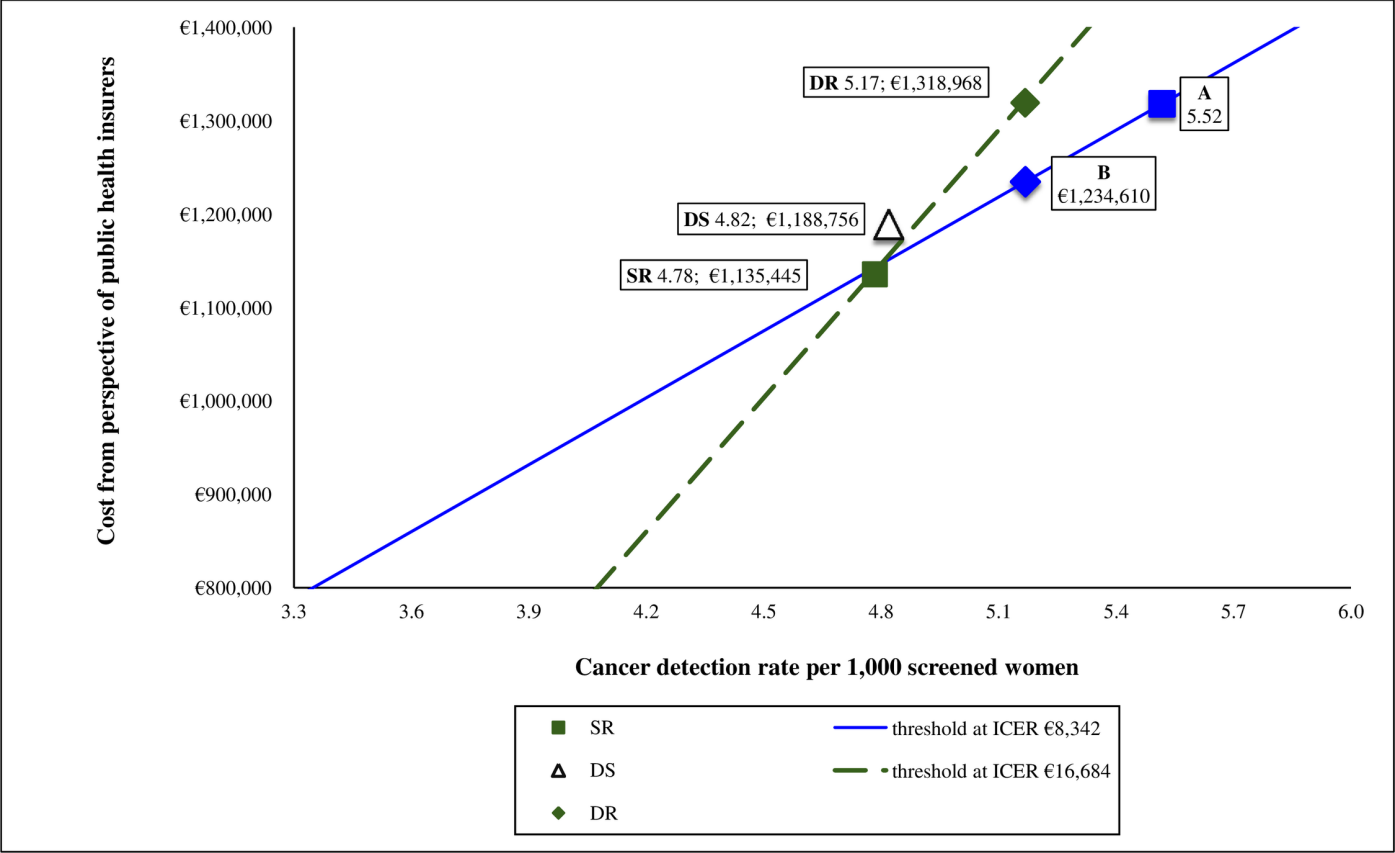
Cost-effectiveness plane illustrating differences in costs and cancer detection rates between the reading strategies. DR = Double reading. SR = Single reading. DS = Double reading in prevalent screening and single reading in incident screening. Continuous and dashed lines represent the thresholds if willingness to pay per one additionally detected cancer were €8,342 and €16,684, respectively. Point A represents the expected cancer detection rate at single reading if willingness to pay per single reading were equal to double reading. Point B represents the expected cost at single reading if the cancer detection rate at single reading were equal to that of double reading.

In Fig 3, the ICERs are plotted over the cost-effectiveness plane where the continuous line represents the expected performance at single reading. The discontinuous line shows that shifting from single to double reading resulted in an increment of €183,523 in cost, and an increase of 0.39‰ in the cancer detection rate. In order to be as cost-effective as single reading, the required cancer detection rate and cost of double reading should shift to points A and B, respectively.

### Sensitivity analysis

**Fig 4:**
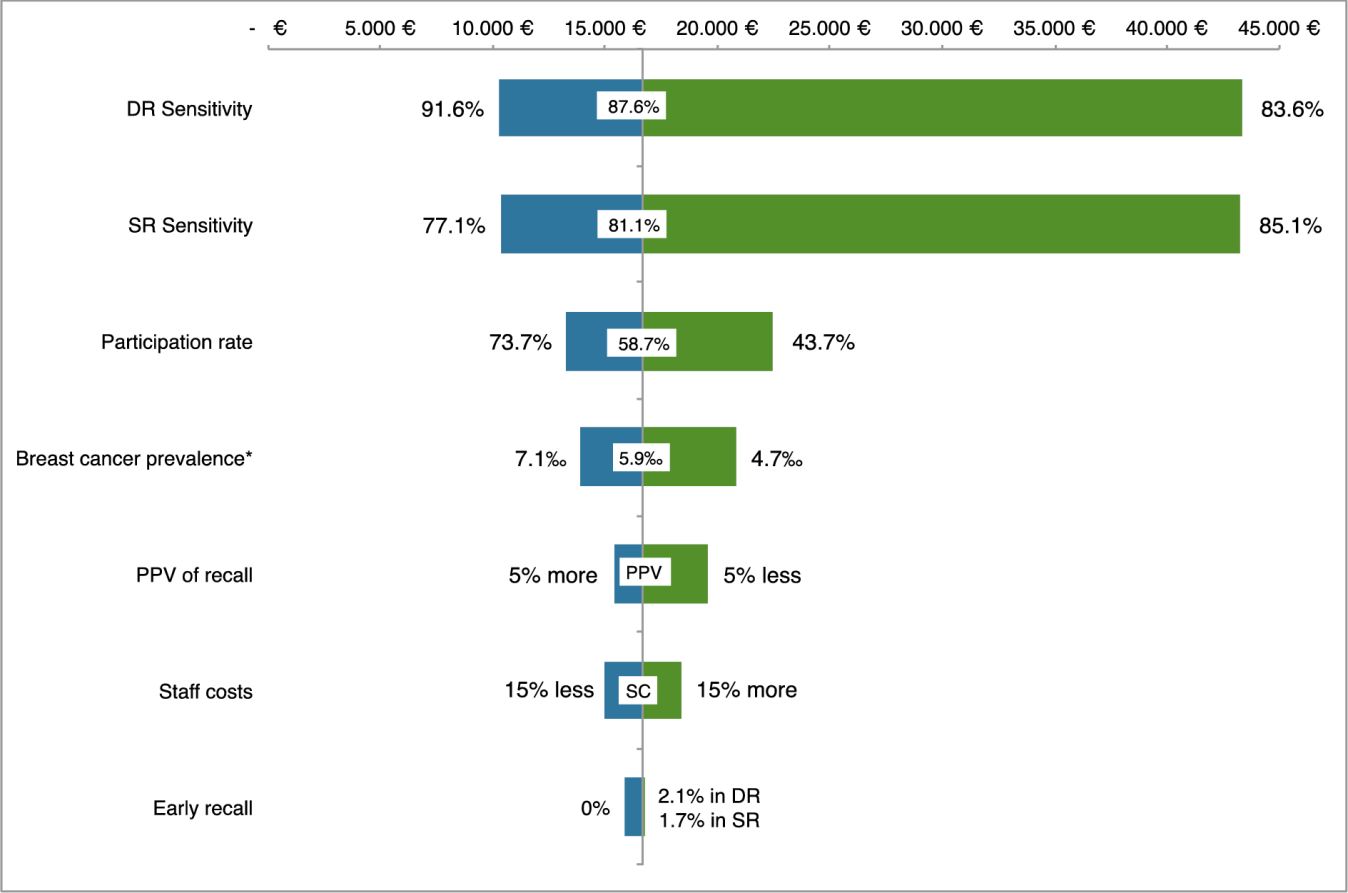
Sensitivity analysis of the incremental cost-effectiveness ratio (ICER) of double reading versus single reading. DR = Double reading. SR = Single reading. PPV = positive predictive value. SC = Staff costs. ER = early recall was 2.1% in double reading and 1.7% in single reading. *The prevalence of breast cancer was estimated as the number of true positives plus the number of false negatives.

Our model was more sensitive to changes in terms of sensitivity of reading strategies for detecting cancers. Varying the current participation rate and observed breast cancer prevalence also affected our results, whereas changes in PPV of recall, staff costs or early recall proportion had less impact on the results (Fig 4 and S5 Table).

## Discussion

We propose a decision-tree model to compare three reading strategies in a population-based breast cancer screening programme. The results of this cost-effectiveness analysis showed that the ICER per one additionally detected cancer was drastically higher (100% more expensive) for double reading than the average cost of each screen detected cancer at single reading. To our knowledge, this is one of the first economic evaluations of double reading versus single reading in the context of digital mammography [18], and the first one assessing single reading without CAD [27].

Our results might be useful for exploring those strategies that require less expensive ICER per one additionally screen detected cancer. Firstly, we found that the combination of double reading in prevalent screening and single reading in incident screening was not cost-effective due to extended dominance, which means that its ICER was higher than that of double reading. This result can be explained by the characteristics of cancers in incident screening that are frequently detected after discordance between readers [14]. Secondly, the effectiveness to detect cancers was similar between double reading and single reading. In fact, an agreement between readers’ interpretations was observed in 94.4% of mammograms and the cancer detection rate of double reading increased only by 8%, as reported in a systematic review [6]. Thirdly, the ICER (€16,684) of double reading was approximately 100% more expensive than the average cost of one screen detected cancer at single reading. Therefore, double reading might not be as cost-effective as single reading.

Aside from the ICER, characteristics of additionally detected cancers after double reading might inform about the potential benefits of this reading strategy in breast cancer screening programmes. European guidelines recommend to limit the proportion of detected ductal carcinomas in-situ (DCIS) [1], due to its potential association with overdiagnosis. On the other hand, there is evidence that DCIS detection reduces interval cancer rates [31]. Based on a Spanish cohort, Blanch et al. [15] reported that double reading had a greater effect on detection of CIS. Similarly, we found that detection of CIS increased by 25% at double reading. Although currently there is not evidence that screening programmes should take measures to reduce DCIS detection, further studies will focus on the association between reading strategies and overdiagnosis.

In our study, both lower recall and early recall rates at single reading show important cost savings due to a reduction in additional diagnostic tests. This net benefit was not at the expense of decreasing its positive predictive value (PPV), which remained stable when comparing it with double reading. Our results match with The US National Cancer Institute findings that showed a better interpretive accuracy and higher PPV at single reading [31].

The ICER at double reading remained rather stable in the sensitivity analysis. However, a small change (5%) in the sensitivity of double reading or single reading could carry important variations in the ICER. To be as cost-effective as single reading, detection rate at double reading should increase by 16%. On the contrary, a reduction in staff cost was not key determinant of ICER variations. Because the parameters we used to test uncertainties reflect the European guidelines’ recommendations [1], we believe that our results might provide reasonable estimates to be extrapolated to other screening programmes with similar characteristics.

In contrast with our results, previous studies comparing double versus single reading reported that double reading was a cost-effective strategy [6,22]. This discrepancy can be explained by the fact that those studies were performed in the context of screen-film mammography. Digital mammography can be more sensitive than screen-film mammography, reducing the possibility of missed lesions. Furthermore, previous studies were performed decades ago in the firsts rounds of screening programmes when costs were probably lower than nowadays. Our results came from the fifth round of a well-established programme when the high experience of one single reader can be as good as the combination of two readers.

Although the scientific evidence is insufficient to determine the benefits of CAD on the readers interpretation [23,32,33]. Sato et al. [24] found that single reading + CAD was a cost-effective strategy increasing life years gained at a ‘low’ price. Our results cannot be comparable with those published by Sato et al. [24] because we did not evaluate CAD and we did not have evidence of life-year gained with single reading. However, the results of both studies may stimulate rethinking of single reading alone or single reading + CAD as a feasible and efficient strategy in settings where shortage of radiologists exists.

The study has certain limitations. First, we did not assess the quality-adjusted life years (QALYs) for each strategy. Although there is no universal agreement, QALY is currently the most appropriate measure of health benefit, and interventions are usually considered cost-effective when the ICER placed on a QALY gained is up to €30,000 [28]. Nevertheless, our results might be useful for calculating expected costs per one additionally screen detected cancer and, therefore, can be taken into account when planning or evaluating breast cancer screening programmes. Second, it is difficult to compare our results with published cost-effectiveness evaluations [18] because of known differences in programmes between countries. However, our results may be similar to other European breast cancer screening programmes. Third, several economic evaluations have reported superiority of single reading with CAD over double reading [23,24]. We did not evaluate this new technology because it is not widely available in Spain. Fourth, although there is no statistical difference in detection rate between single and double reading, this study may not be adequately powered for the small expected difference. Finally, in this study we included information about five highly trained radiologists. The results can be different in other context with less trained radiologists or other professionals performing the reading of mammograms.

In conclusion, our results suggest that, in the context of digital mammography, double reading double reading can be as effective as single reading but more expensive. Whereas double reading did not dramatically increase the cancer detection rate, the current question is how much the decision makers are willing to pay for the extra cancers detected. Further economic evaluation of randomised controlled trials may be crucial in determining whether the QALYs gained at double reading are comparable to those at single reading in the context of digital mammography.

## Supporting Information

S1 Table Definitions of performance measure and diagnostic accuracy outcomes. (DOCX)

S2 Table Unitary costs and cost estimation according to reading strategy. NA = not apply. ^¶^Staff **=** medical, technician, and administrative. *Maintenance and repairs of the machine including depreciation. ^ß^Administrative staff in early recall, as well as in consensus and arbitration. ^∑^In cancers cases suspected by one or both readers but with missing data, the average cost of additional tests was imputed.
(DOCX)

S3 Table Value of parameters used in the sensitivity analysis. (DOCX)

S4 Table Characteristics of cancers detected at each reading strategy. ^¶^Detection rate per 1000 screened women. ^‡^Linear test. ^§^Interval cancers detected in the subsequent two years after a negative screening episode.
(DOCX)

S5 Table Results of the sensitivity analysis. FN = false negatives.
(DOCX)
