# Supplementary material for: Cost-Effectiveness of Double Reading versus Single Reading of Mammograms in a Breast Cancer Screening Programme
Source: PLoS One. 2016 Jul 26;11(7):e0159806. doi: 10.1371/journal.pone.0159806 (PMC4961365; doi:10.1371/journal.pone.0159806)
Supplement: S1 Table — (DOCX) [file pone.0159806.s001.docx]

| **Outcome** | **Definitions** |
| --- | --- |
| **Performance measure** |  |
| Concordance between radiologists | It was considered as a concordant mammography result if both radiologists made the same classification: (1) recall; (2) rarly recall; or (3) two-year screening. |
| Positive predictive value (PPV) of recall | Number of detected breast cancers divided by the number of women recalled for additional tests. |
| Interval cancers (IC) | Breast cancers detected during the subsequent two years after the screening mammogram was performed. Cancers detected in ‘Early recall’ or correctly suspected by radiologists, but missed in additional tests, were not considered as interval cancers. |
| **Diagnostic accuracy** |  |
| Screen detected cancers | Breast cancers diagnosed in mammograms classified as ‘Recall’. Invasive cancers as well as non-invasive ductal carcinomas in situ were included. Cancers with non-breast histology were excluded. |
| Cancer detection rate | Number of true-positives divided by the number of participating women. |
| False-positives (FP) | Mammograms classified as ‘Recall’ without any cancer confirmation after additional tests. |
| True-negatives (TN) | Mammograms classified as ‘Two-year screening’ without any cancer detection during the subsequent two years. |
| False-negatives (FN) | False-negatives were cancers with screening-mammograms classified as ‘Two-year screening’. During a 2-year follow-up, until June 2013, all mammography-reports corresponding to interval cancers were retrospectively evaluated to distinguish, when possible, between true interval cancers and FN. FN were defined as interval cancers that had shown screening-mammograms with minimal signs. |
| Sensitivity (%) | p positive test  (screen detected cancers+ FN)  p= percentage; test += mammography classified as ‘Recall’ |
| Specificity (%) | p test -  (TN+ FP)  p= percentage; test += mammography classified as ‘Two-year screening’ |
