# Supplementary material for: Cost-Effectiveness of Double Reading versus Single Reading of Mammograms in a Breast Cancer Screening Programme
Source: PLoS One. 2016 Jul 26;11(7):e0159806. doi: 10.1371/journal.pone.0159806 (PMC4961365; doi:10.1371/journal.pone.0159806)
Supplement: S2 Table — NA = not apply. ¶Staff = medical, technician, and administrative. *Maintenance and repairs of the machine including depreciation. ßAdministrative staff in early recall, as well as in consensus and arbitration. ∑In cancers cases suspected by one or both readers but with missing data, the average cost of additional tests was imputed. (DOCX) [file pone.0159806.s002.docx]

| **Common costs for all three reading strategies** | **Unitary cost** |  | | | | | |
| --- | --- | --- | --- | --- | --- | --- | --- |
|  |  | **No.** | | | **Cost €** | | |
| ^¶^Staff | NA | NA | | | 690,510.20 | | |
| *Maintenance | NA | NA | | | 55,177.88 | | |
| Accessories, supplies, post-mail | 2.47 | 28,636 | | | 70,702.10 | | |
| Mammography and radioprotection | 1.36 | 28,636 | | | 38,907.33 | | |
| **Non-common costs according to reading strategies** |  | **Double reading** | | **Double reading in prevalent screening and single reading in incident screening** | | **Single reading** | |
|  |  | **No.** | **Cost €** | **No.** | **Cost €** | **No.** | **Cost €** |
| Mammography in early recall | 5.15 | 609 | 3,137.70 | 523 | 2,694.60 | 490 | 2524.59 |
| **Staff** |  |  |  |  |  |  |  |
| Readings | 3.48 | 58,490 | 203,704.30 | 35,419 | 123 354.47 | 29,126 | 101,437.71 |
| Consensus | 6.97 | 1,671 | 11,639.25 | 692 | 4,820.08 | 0 | 0.00 |
| Arbitration | 10.45 | 30 | 313.45 | 4 | 41.79 | 0 | 0.00 |
| ^ß^Administrative (out of fixed costs) | 9.02 | 3,724 | 33,597.64 | 2,595 | 23,411.89 | 1,851 | 16,699.58 |
| Technician in early recall | 10.03 | 609 | 6,108.19 | 523 | 5,245.62 | 490 | 4,914.64 |
| **Additional tests to confirm or ruled out malignancy** | | | | | | | |
| No. of recalled women |  | 1,414 |  | 1,376 |  | 1361 |  |
| Ultrasound | 50.14 | 1,252 | 62,775.28 | 1,178 | 59,064.92 | 1,121 | 56,206.94 |
| Mammography (out of screening) | 32.67 | 831 | 27,148.77 | 778 | 25,417.26 | 760 | 24,829.20 |
| Fine-needle aspiration cytology | 141.82 | 253 | 35,880.46 | 230 | 32,618.60 | 202 | 28,647.64 |
| Core biopsy | 131.77 | 255 | 33,601.35 | 194 | 25,563.38 | 154 | 20,292.58 |
| Open surgical biopsy | 1,536.00 | 16 | 24,576.00 | 11 | 16,896.00 | 9 | 13,824.00 |
| Other diagnostic tests (average) | 194.85 | 106 | 20,653.57 | 71 | 13,834.00 | 53 | 10,326.79 |
| ^∑^Missing imputation | Average | 2 | 534.12 | 2 | 495.91 | 2 | 443.29 |
| **Total** |  | **1,318,967.60** | | **1,188,756.04** | | **1,135,444.46** | |
