# Supplementary material for: Cost-Effectiveness of Double Reading versus Single Reading of Mammograms in a Breast Cancer Screening Programme
Source: PLoS One. 2016 Jul 26;11(7):e0159806. doi: 10.1371/journal.pone.0159806 (PMC4961365; doi:10.1371/journal.pone.0159806)
Supplement: S3 Table — (DOCX) [file pone.0159806.s003.docx]

|  | **Range of variation** | |  | |
| --- | --- | --- | --- | --- |
| **Parameter** | **High value** | **Low value** | **Reason** |  |
| Percentage of participation | 74% | 44% | >70% is recommended by European guidelines.  45% was the lowest participation rate observed in the Programme. |  |
| Prevalence of breast cancer (percentage of participants who were identified as true positives or as false negatives) | (Observed) -20% | (Observed) +20% | This range includes the observed prevalence during the second to fourth round of the Programme. |  |
| Sensitivity of reading strategy | >70% in sensitivity per each reading strategy. On the other hand, in order to evaluate the consequences of switch from the first to the second reader, we included the sensitivity showed by the second radiologist rather than the first one. | | Although there are not specific standards recommended in European guidelines, expected sensitivity for a reader in a screening Programme might be superior to 70% | |
| Positive-predictive value (PPV) of recall | 5% | 10% | Without changes in the number of detected cancers, a higher recall rate entails a higher number of unnecessary diagnostic tests. Then, a reverse association between the PPV of recall and the costs attributable to diagnostic tests was expected. We changed the PPV between 5% and 10%, as there is not great uncertainty around this percentage. As a consequence, costs changed according with the variation of the PPV. |  |
| Staff costs | (Observed) -15% | (Observed) +15% | There is not great uncertainty around this percentage. |  |
| Early recall rate | 0% | 0% | A lowest proportion of early recall is recommended (<0.5%) by the European guidelines |  |
