# Supplementary material for: Cost-Effectiveness of Double Reading versus Single Reading of Mammograms in a Breast Cancer Screening Programme
Source: PLoS One. 2016 Jul 26;11(7):e0159806. doi: 10.1371/journal.pone.0159806 (PMC4961365; doi:10.1371/journal.pone.0159806)
Supplement: S4 Table — ¶Detection rate per 1000 screened women. ‡Linear test. §Interval cancers detected in the subsequent two years after a negative screening episode. (DOCX) [file pone.0159806.s004.docx]

|  | **Double reading** | | **Double reading in prevalent screening and single reading in incident screening** | | **Single reading** | |  | **Additionally detected cancers at double compared to single reading** | |
| --- | --- | --- | --- | --- | --- | --- | --- | --- | --- |
|  | **No.** | **%** | **No.** | **%** | **No.** | **%** | P value | **No.** | **%** |
| Total cancer detection rate^¶^ | 148 | 5.17‰ | 138 | 4.82‰ | 137 | 4.78‰ | .768 | 11 | 0.38‰ |
| Prevalent screening^¶^ | 44 | 7.36‰ | 44 | 7.36‰ | 43 | 7.19‰ | .918 | 1 | 0.17‰ |
| Incident screening^¶^ | 104 | 4.59‰ | 94 | 4.15‰ | 94 | 4.15‰ |  | 10 | 0.44‰ |
| Interval cancers^§^ | 16 | 0.56‰ | 26 | 0.91‰ | 27 | 0.94‰ | .200 | -11 | -0.39‰ |
| **Age at screening** |  |  |  |  |  |  |  |  |  |
| 50-54 | 46 | 31.3 | 45 | 32.6 | 45 | 32.8 | .981^‡^ | 1 | 2.3 |
| 55-59 | 39 | 26.4 | 34 | 24.6 | 33 | 24.1 |  | 6 | 18.2 |
| 60-64 | 32 | 21.6 | 29 | 21.0 | 29 | 21.2 |  | 3 | 10.3 |
| 65-69 | 31 | 20.9 | 30 | 21.7 | 30 | 21.9 |  | 1 | 3.4 |
| **Histologic type** |  |  |  |  |  |  |  |  |  |
| Invasive | 116 | 79.5 | 111 | 81.6 | 111 | 82.2 | .822 | 5 | 4.5 |
| In situ | 30 | 20.5 | 25 | 18.4 | 24 | 17.8 |  | 6 | 25.0 |
| Unknown | 2 | - | 2 | - | 2 | - |  |  |  |
| **Stage** |  |  |  |  |  |  |  |  |  |
| In situ | 30 | 20.5 | 25 | 18.4 | 24 | 17.8 | .781^‡^ | 6 | 25.0 |
| I | 75 | 51.4 | 72 | 52.9 | 72 | 53.3 |  | 3 | 4.2 |
| IIA | 25 | 17.1 | 25 | 18.4 | 25 | 18.5 |  | 0 | 0 |
| IIB | 12 | 8.2 | 10 | 7.4 | 10 | 7.4 |  | 2 | 20.0 |
| IIIA | 1 | 0.7 | 1 | 0.7 | 1 | 0.7 |  | 0 | 0 |
| IIIB | 2 | 1.4 | 2 | 1.5 | 2 | 1.5 |  | 0 | 0 |
| IV | 1 | 0.7 | 1 | 0.7 | 1 | 0.7 |  | 0 | 0 |
| Unknown | 2 | - | 2 | - | 2 | - |  |  |  |
| **BIRADS** |  |  |  |  |  |  |  |  |  |
| 0 | 66 | 44.9 | 62 | 44.9 | 58 | 42.3 | .922 | 10 | 17.9 |
| 3 | 6 | 4.1 | 6 | 4.3 | 6 | 4.4 |  | 1 | 20.0 |
| 4 | 9 | 6.1 | 4 | 2.9 | 7 | 5.1 |  | 0 | 0 |
| 5 | 66 | 44.9 | 66 | 47.8 | 66 | 48.2 |  | 0 | 0 |
| **Mammographic features** |  |  |  |  |  |  |  |  |  |
| Mass | 71 | 49.3 | 68 | 50.0 | 67 | 49.6 | 1.000 | 4 | 5.9 |
| Calcifications | 33 | 22.9 | 29 | 21.3 | 29 | 21.5 |  | 4 | 14.3 |
| Mass with calcifications | 25 | 17.4 | 25 | 18.4 | 25 | 18.5 |  | 0 | 0 |
| Asymmetric density | 6 | 4.2 | 6 | 4.4 | 6 | 4.4 |  | 0 | 0 |
| Architectural distortion | 9 | 6.2 | 8 | 5.9 | 8 | 5.9 |  | 1 | 12.5 |
| Unknown | 4 | - | 2 | - | 2 | - |  | 2 | - |
| **Tumour size** |  |  |  |  |  |  |  |  |  |
| ≤10 mm | 55 | 37.7 | 50 | 36.8 | 49 | 36.3 | .971 | 6 | 12.2 |
| >10 mm | 91 | 62.3 | 86 | 63.2 | 86 | 63.7 |  | 5 | 5.6 |
| Unknown | 2 | - | 2 | - | 2 | - |  |  |  |
| **Lymph node involved** |  |  |  |  |  |  |  |  |  |
| Yes | 32 | 21.6 | 31 | 22.5 | 31 | 22.6 | .976 | 1 | 3.2 |
| **Molecular subtype** |  |  |  |  |  |  |  |  |  |
| Luminal A | 63 | 56.3 | 59 | 56.7 | 59 | 56.7 | 1.000 | 4 | 6.9 |
| Luminal B | 11 | 9.8 | 11 | 10.6 | 11 | 10.6 |  | 0 | 0 |
| HER 2 | 28 | 25.0 | 24 | 23.1 | 24 | 23.1 |  | 4 | 16.6 |
| Triple negative | 10 | 8.9 | 10 | 9.6 | 10 | 9.6 |  | 0 | 0 |
| Unknown | 35 | - | 34 | - | 33 | - |  | 3 | - |
