# Supplementary material for: Cost-Effectiveness of Double Reading versus Single Reading of Mammograms in a Breast Cancer Screening Programme
Source: PLoS One. 2016 Jul 26;11(7):e0159806. doi: 10.1371/journal.pone.0159806 (PMC4961365; doi:10.1371/journal.pone.0159806)
Supplement: S5 Table — FN = false negatives. (DOCX) [file pone.0159806.s005.docx]

| **Parameters** | **Expected cost €** | **Cancer detection rate** | **No. of cancers** | **Incremental cost €** | **Incremental effect** | **Average cost €** | **Variations in the ICER €** | |
| --- | --- | --- | --- | --- | --- | --- | --- | --- |
| **Sensitivity of double reading** | | | | | | | |  |
| **83.6%** |  |  |  |  |  |  |  | |
| **Single reading** | 1,135,444.46 | 4.78 | 137 |  |  | 8,287.92 |  | |
| **Double reading** | 1,318,967.60 | 4.91 | 141 | 183,523.14 | 4 | 9,338.48 | 43,283.76 | |
| **91.6%** |  |  |  |  |  |  |  | |
| **Single reading** | 1,135,444.46 | 4.78 | 137 |  |  | 8,298.09 |  | |
| **Double reading** | 1,318,967.60 | 5.43 | 155 | 183,152.80 | 18 | 8,522.66 | 10,312.66 | |
| **Sensitivity of single reading** | | | | | | | |  |
| **77.1%** |  |  |  |  |  |  |  | |
| **Single reading** | 1,135,444.46 | 4.54 | 130 |  |  | 8,718.09 |  | |
| **Double reading** | 1,318,967.60 | 5.17 | 148 | 183,523.14 | 18 | 8,911.94 | 10,333.51 | |
| **85.1%** |  |  |  |  |  |  |  | |
| **Single reading** | 1,135,444.46 | 5.02 | 144 |  |  | 8,298.09 |  | |
| **Double reading** | 1,318,967.60 | 5.17 | 148 | 183,152.80 | 4 | 8,911.94 | 43,196.42 | |
| **Participation rate** | | | | | | | |  |
| **43.7%** |  |  |  |  |  |  |  | |
| **Single reading** | 1,135,444.46 | 4.78 | 102 |  |  | 11,134.25 |  | |
| **Double reading** | 1,318,967.60 | 5.17 | 110 | 183,523.14 | 8 | 11,972.59 | 22,413.72 | |
| **73.7%** |  |  |  |  |  |  |  | |
| **Single reading** | 1,135,444.46 | 4.78 | 172 |  |  | 6,600.56 |  | |
| **Double reading** | 1,318,967.60 | 5.17 | 186 | 183,523.14 | 14 | 7,097.54 | 13,287.21 | |
| **Breast cancer prevalence (screen detected cancers + FN)** | | | | | | | |  |
| **4.7/1000** |  |  |  |  |  |  |  | |
| **Single reading** | 1,135,444.46 | 3.83 | 110 |  |  | 10,359.89 |  | |
| **Double reading** | 1,318,967.60 | 4.13 | 118 | 183,523.14 | 9 | 11,139.93 | 20,854.90 | |
| **7.1/1000** |  |  |  |  |  |  |  | |
| **Single reading** | 1,135,444.46 | 5.74 | 164 |  |  | 6,906.60 |  | |
| **Double reading** | 1,318,967.60 | 6.20 | 178 | 183,523.14 | 13 | 7,426.62 | 13,903.97 | |
| **PPV of recall** |  |  |  |  |  |  |  | |
| **5% less (Double reading =5.2%, Single reading =4,8%)** | | | | | | | |  |
| **Single reading** | 1,292,580.51 | 4.78 | 137 |  |  | 9,434.89 |  | |
| **Double reading** | 1,507,464.00 | 5.17 | 148 | 214,883.49 | 11 | 10,185.57 | 19,534.86 | |
| **5% more (Double reading =15.2%, Single reading =14,8%)** | | | | | | | |  |
| **Single reading** | 1,084,783.98 | 4.78 | 137 |  |  | 7,918.13 |  | |
| **Double reading** | 1,254,084.29 | 5.17 | 148 | 169,300.31 | 11 | 8,473.54 | 15,390.94 | |
| **Staff costs** |  |  |  |  |  |  |  | |
| **15% less (Double reading = €788,970.94, Single reading = €681,843.10)** | | | | | | | |  |
| **Single reading** | 1,015,119.20 | 4.78 | 137 |  |  | 7,409.63 |  | |
| **Double reading** | 1,179,737.43 | 5.17 | 148 | 164,618.23 | 11 | 7,971.20 | 14,965.29 | |
| **15% more (Double reading = €1,067,431.27, Single reading = €922,493.61)** | | | | | | | |  |
| **Single reading** | 1,255,769.71 | 4.78 | 137 |  |  | 9,166.20 |  | |
| **Double reading** | 1,458,197.76 | 5.17 | 148 | 202,428.05 | 11 | 9,852.69 | 18,402.55 | |
| **Early recall reduced to 0%** | | | | | | | |  |
| **Single reading** | 1,116,484.53 | 4.50 | 129 |  |  | 8,654.92 |  | |
| **Double reading** | 1,290,868.24 | 4.89 | 140 | 174,383.71 | 11 | 9,220.49 | 15,853.06 | |
